# Primary retroperitoneal extraovarian granulosa cell tumor

**DOI:** 10.4322/acr.2021.355

**Published:** 2022-02-11

**Authors:** Pragya Sharma, Vikram Singh, Niharika Mishra, Manoj Gopinath, Prashant Gupta

**Affiliations:** 1 Army Hospital Research and Referral, Department of Lab Sciences and Molecular Medicine, New Delhi, India; 2 Army Hospital Research and Referral, Department of Radiodiagnosis, New Delhi, India; 3 Army Hospital Research and Referral, Department of Surgical Oncology, New Delhi, India

**Keywords:** Granulosa cell tumor, immunohistochemistry, inhibins

## Abstract

Extraovarian granulosa cell tumors (GCTs) develop from ectopic gonadal tissue situated along the embryonal route of the genital ridge. Primary retroperitoneal tumors are extremely rare, with an incidence of 02% -06% and 80-85% probability of malignancy. Only eight such case reports have been published previously. We herein, report a rare case of extraovarian retroperitoneal GCT in a 55-year-old woman who presented with intermittent left lumbar region pain of one-year duration. She had a history of hysterectomy and bilateral salpingo-oophorectomy 8 years ago for uterine leiomyoma. Laparotomy revealed a retroperitoneal mass measuring 8cm x 10cm x 20cm in size, solid cystic with areas of necrosis and hemorrhage. The gross features, classical histopathology, and positive immunostaining of the retroperitoneal mass with inhibin, calretinin, PR, WT1 and immunonegativity for EMA were characteristic of adult-type GCT. Excluding any previous history of primary ovarian GCT in this patient, a de-novo retroperitoneal diagnosis was established.

## INTRODUCTION

Granulosa cell tumors (GCT) of the ovary are rare and represent 2% - 5% of all ovarian neoplasms^1^. GCTs are sex cord-gonadal stromal or non-epithelial group of tumors that are composed of granulosa cells, theca cells, and fibroblasts in varying proportions and combinations.^2^ They can recur or metastasize many years after initial treatment and can rarely develop at an extraovarian site, even in an oophorectomized patient.^3^ Primary GCTs occurring at extraovarian sites are rare, and having a primary tumor arising from the retroperitoneum is exceedingly rare.^4^ We present an unusual case of extraovarian retroperitoneal GCT in a postmenopausal woman. This case is reported for its rarity, ectopic presentation, and to describe its relevance to the embryonic origin.

## CASE REPORT

A 55-year-old woman with no comorbidities presented with intermittent pain in the left lumbar region of one year duration. There was no associated history of weight loss, fever, or urinary complaints. Per abdominal examination revealed an 8cm x10cm ill-defined non tender and non-mobile mass in left lumbar region extending superiorly into left hypochondrium and inferiorly into pelvis. The patient had undergone total abdominal hysterectomy with bilateral salpingo-oophorectomy 8-years ago for uterine leiomyoma at our center with no evidence of primary GCT of the ovaries on histopathological evaluation. The slides of previous surgery were retrieved from department achieves, and both ovaries showed normal histomorphology on review.

**Figure 1:**
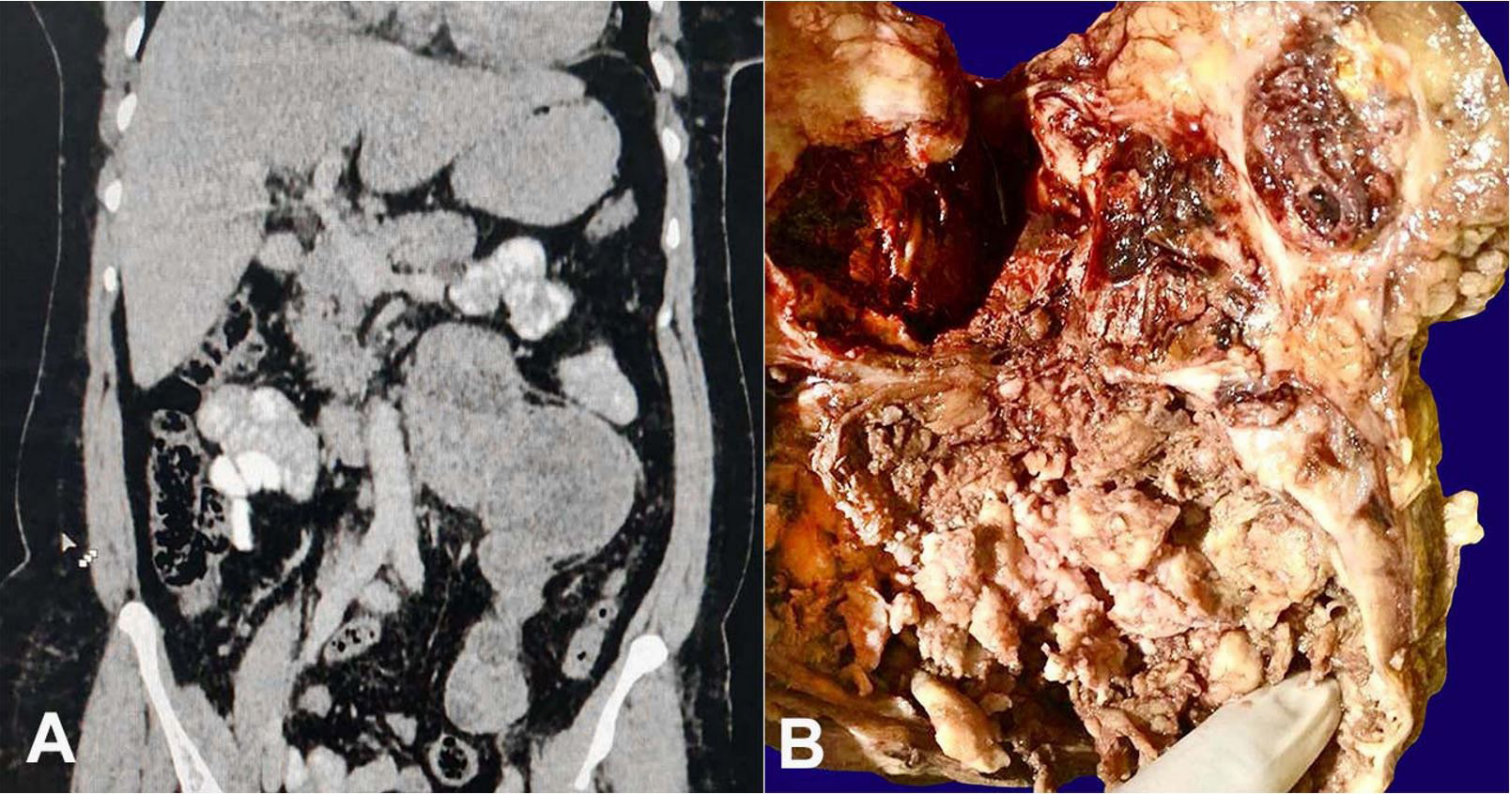
A – Computed tomography scan (coronal view) showing a longitudinally oriented, heterogeneously enhancing solid cystic retroperitoneal mass; B – Gross view of the solid cystic mass with areas of hemorrhage and necrosis. Note clotted blood within the cysts.

Abdominal-pelvic computer tomography imaging showed a longitudinally oriented heterogeneously enhancing solid cystic retroperitoneal mass measuring 7.2cm x 10.3cm x 19.4cm epicentered near the left kidney (Figure 1A). The mass was compressing the left renal vein superiorly, extending up to left psoas inferiorly, abutting the aorta medially and left renal pelvis laterally, displacing the bowel loops anteriorly and abutting the left ureter posteriorly. The left kidney showed features of hydronephrosis. Uterus and bilateral adnexa were not visualized. The laboratory investigations including metanephrines, normetanephrines, chromogranin, CA -125, CEA, AFP and beta HCG levels were within normal biological range. The patient underwent laparotomy and excision of the retroperitoneal mass. On gross examination, the mass weighed 450gm and measured 8cm x 10cm x 20cm. It was partially encapsulated. The cut surface was solid cystic showing areas of hemorrhage and necrosis. The cysts were filled with clotted blood (Figure 1B).

**Figure 2:**
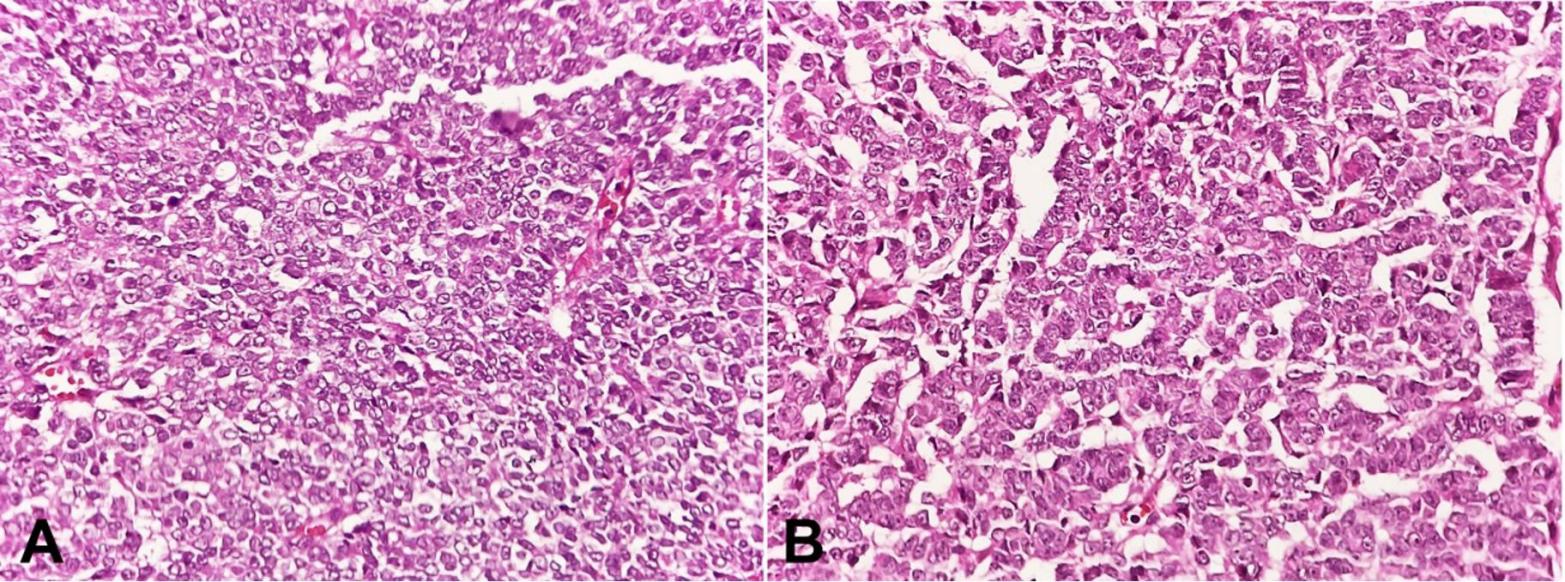
A - Photomicrograph of the tumor showing small uniform tumor cells with scant cytoplasm and round to oval nuclei exhibiting nuclear grooves and arranged in a diffuse pattern (H&E, 40X); B – tumor cells arranged in a trabecular pattern (H&E, 40X).

Microscopic findings showed neoplastic cells arranged in diffuse, pseudopapillary, macrofollicular, microfollicular, and gyriform patterns. The tumor cells were small, uniform with scant cytoplasm, and round to oval nuclei exhibiting nuclear grooves (Figure 2).

**Figure 3:**
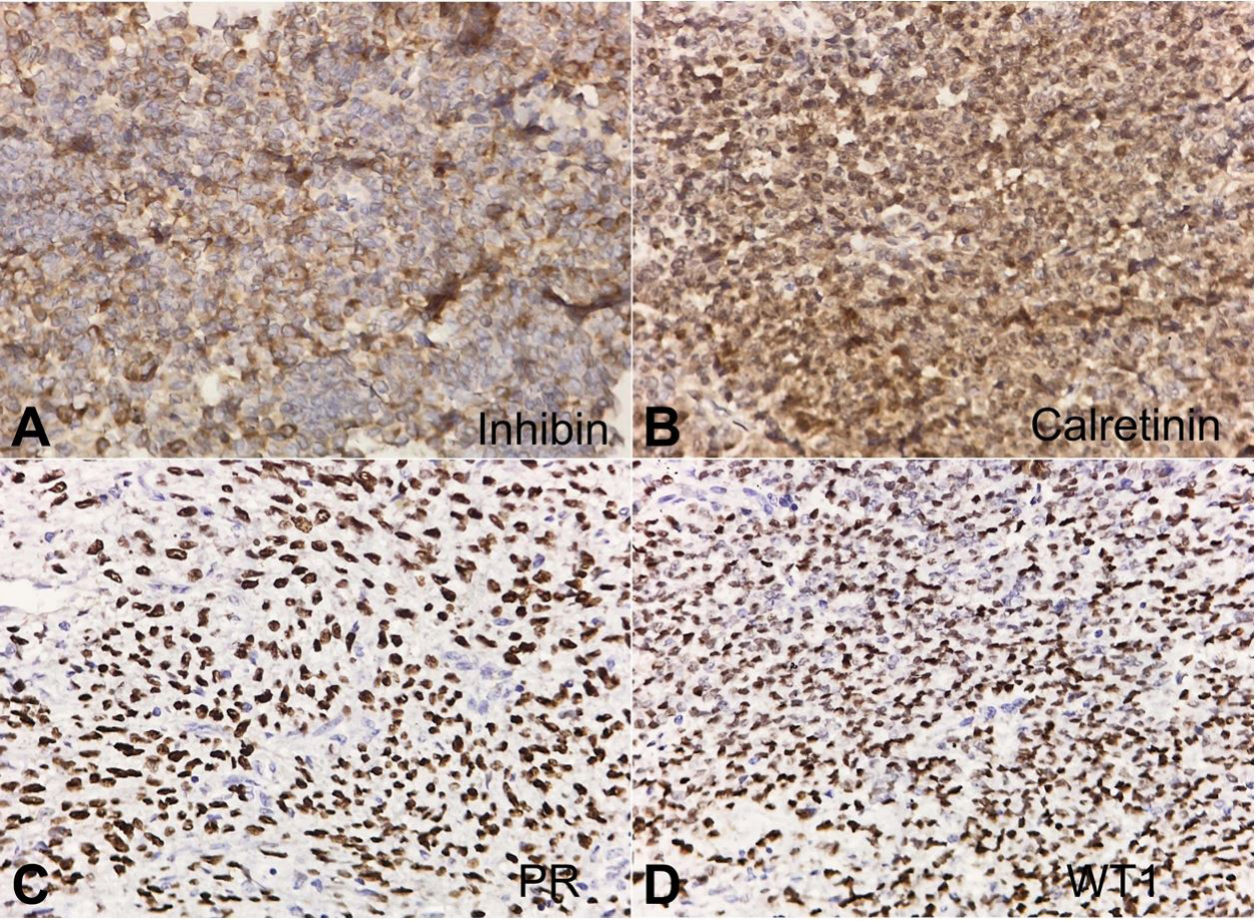
A– Cytoplasmic inhibin positivity in tumor cells (40X); B – Nuclear and cytoplasmic calretinin positivity in tumor cells (40X); C– Nuclear PR positivity in tumor cells (40X). D – Nuclear WT1 positivity in tumor cells (40X).

Call-Exner bodies were present. Lymphovascular space invasion was identified. On performing immunohistochemistry (IHC), the tumor cells were immunopositive for inhibin (Figure 3A), calretinin (Figure 3B), PR (Figure 3C), WT1 (Figure 3D) and negative for EMA. Considering the location, typical histomorphology and immunoprofile with absence of past history of GCT, the lesion was diagnosed as extraovarian GCT, adult type.

The patient’s intraoperative and postoperative courses were uneventful.

## DISCUSSION

Granulosa cell tumors (GCTs) are malignant sex cord— stromal tumors of the ovary. These can be of adult or juvenile type based on clinical and histopathological features. The adult type of GCTs is more common and usually present during the perimenopausal or the early menopausal period, the median age being 50–54 years.^3^ Patients with GCT require long-term follow-up with history, clinical examination, and tumor marker studies because 17% of relapses occur more than 10 years after diagnosis.^3^ The pelvis is the most common site of recurrence. Infrequently, GCTs can develop at extra-ovarian sites and have been documented to develop in the broad ligament, retroperitoneum, mesentery, liver, adrenals,^4^ omentum^5^ and fallopian tubes.^6^ The possibility of metastasis has to be excluded before making a diagnosis of extraovarian GCT. We conducted an electronic search of PubMed and Google Scholar for all original articles and case reports on Adult GCT using the keywords ‘extraovarian granulosa cell tumor’ in combination with ‘retroperitoneum’ and ‘immunohistochemistry’. Out of 11,300 displayed articles, we excluded all the duplicates, misclassified articles, non-pathology articles, articles without abstract or full text, articles on Juvenile GCT, and articles on extraovarian recurrence of primary GCT of the ovary. As a result, we obtained around 30 case reports. After a systematic review of abstract and/or full text, we further excluded studies that did not describe histopathology and IHC in detail and excluded those studies where primary ovarian GCT was not ruled out completely. We also reviewed the references of the articles to not miss out a study. Thus, we were left with 13 relevant case reports that altogether reported primary extraovarian GCT, including retroperitoneal GCT. The comparison of clinical, imaging, and histological findings in reported cases of primary extraovarian GCT is summarized in Table 1.

**Table 1:** Comparison of the reported cases of Primary extraovarian granulosa cell tumor

ref	Age (Years)	Site	Size (cm)	Imaging	Histology	IHC	Ovaries examined & uninvolved
2	50	RP	6	Non-homogenous, multi-septate cystic mass	AGCT	NA	Yes
3	58	RP	16	Well-defined heterogenous mass with areas of necrosis	AGCT	Inhibin + EMA -	Uninvolved as per previous HP report of TAH-BSO
4	52	RP	8	Solid cystic mass	AGCT	Inhibin +, vimentin +, calretinin +, EMA -	Yes
5	55	Omentum	7	Solid cystic mass	AGCT	Inhibin +, PANCK -	Uninvolved as per previous HP report of TAH-BSO
6	62	Left FT	6	Complex cystic mass	AGCT	Inhibin + Calretinin + EMA -	Yes
7	54	RP	8.8	Large lobulated solid mass with necrosis	AGCT	Inhibin + Vimentin + EMA -	Yes
8	60	RP	11	Well-defined cystic mass	AGCT	Inhibin + EMA -	Not examined
9	45	RP		Solid cystic mass	AGCT	Inhibin +	Yes
10	69	RP	12	Solid heterogeneous mass lesion	AGCT	Inhibin + EMA -	Post TAH- BSO status. HP report NA
11	64	RP	13	Large, lobulated, heterogenous mass with cystic components	AGCT	Inhibin +	Uninvolved as per previous HP report of TAH-BSO
13	54	Mesentery	13	Solid heterogeneous mass	AGCT	Inhibin + EMA -	Yes
14	58	Broad ligament	11	Large pelvic tumor	AGCT	Inhibin + Calretinin+ CK 7	Yes
15	63	Adrenal	9	Right suprarenal mass	AGCT	NA	Yes

AGCT: Adult granulomas cell tumor, cm: centimeter, EMA: Epithelial membrane antigen, FT: Fallopian Tube, HP: Histopathology, IHC: Immunohistochemistry, NA: not available, PANCK: pancytoketatin, RP: Retroperitoneum, Ref: reference, TAH-BSO: Total abdominal hysterectomy bilateral salpingo oophorectomy, CK 7: Cytokerartin 7.

Primary retroperitoneal GCTs are very rare, with 02% -06% incidence with 80-85% probability of malignancy.^2^ On extensive literature review, only eight such cases have been reported previously, of which three have been reported from the Indian subcontinent.^2^^,^ 3 ^,^ 4 ^,^ 7 ^-^ 11 A dual origin from both the mesonephros and coelomic epithelium has been suggested. The mesonephros appears to be fundamental for the development of the sex cord. This might suggest the development of GCTs in the retroperitoneum, the broad ligament, or the adrenal, all of which differentiate in close proximity to the mesonephros and the mesonephric duct.^4^

Macroscopically, GCTs vary in size with an average diameter of 10cm. They are solid-cystic and rarely may be solid or entirely cystic. The solid areas are soft tan to yellow, while the cysts typically contain clotted blood. Rupture is associated with areas of hemorrhage.^12^ Microscopically, a variety of architectural patterns occur and are often admixed, including diffuse, microfollicular, macrofollicular, trabecular, nested, pseudopapillary, and gyriform.^12^ Call-Exner bodies are present in 30–60% of the cases. The tumor cells are typically uniform round to oval with scant pale cytoplasm and characteristic ‘coffee bean‘ nuclei. A variable amount of fibromatous or thecomatous stroma may be seen.^12^ Mitosis is variable. Extraovarian GCT should be differentiated from other metastatic carcinomas of the ovary that have similar histomorphology. Inhibin and EMA help in differentiating the tumors and establishing the diagnosis. GCT is positive for inhibin and negative for EMA.^13^ It should also be differentiated from other tumors such as undifferentiated carcinoma, small cell carcinoma, endometrial stromal sarcoma, carcinoid, and lymphomas.^13^ These tumors do not show positivity for inhibin. Immunohistochemistry (IHC) for CK, EMA, CD99, LCA, and chromogranin can help in diagnosing and differentiating these tumors. GCT does not show positivity for EMA, LCA, and chromogranin.^13^ Serum inhibin levels are elevated in GCT thus, inhibin can be used as a marker for GCT.^14^Lappohn et al.^15^ was the first to demonstrate the value of inhibin as a marker for both primary and recurrent disease and showed that a rise in inhibin level preceded clinical recurrence as early as 20 months. Studies using subunit specific ELISA show inhibin B to be the major form secreted in GCT, and that inhibin B is more accurate than inhibin A in detecting GCT.^14^ Studies have further confirmed that inhibin B is a sensitive and specific marker for GCTs.^16^^,^ 17 Thus, inhibin B levels can be helpful in the early detection and recurrence of GCTs.

In our present case, GCT was not suspected clinically or radiologically and the preoperative tumor markers were within normal biological range. The diagnosis was made by characteristic histopathological features and confirmed by immunopositivity for inhibin, calretinin, PR, WT1, and immunonegativity for EMA.

## CONCLUSION

Granulosa cell tumors can arise in locations other than the ovary and are considered to be derived from the mesenchyme of the genital ridge. Women who have undergone oophorectomy may develop GCT at extraovarian location, including retroperitoneum. Thus, GCT should be kept as a differential diagnosis of retroperitoneal masses in female patients. Immunohistochemistry helps in differentiating GCT from other neoplasms.
